# College and Hospital Notes

**Published:** 1907-11

**Authors:** 


### COLLEGE AND HOSPITAL NOTES.

THE merger between the Louisville Medical College and the Hospital College of Medicine, according to the Journal of the American Medical Association, has finally been completed and the college will henceforth be known as the Louisville and Hospital Medical College. It will be the medical department of Central University. The officers of the new college are as follows: Dr. L. S. McMurtry, president; Dr. C. W. Kelly, dean; Drs. H. B. Ritter and H. H. Grant, regents; Dr. Irving Abell, secretary of the faculty. This merger is most commendable and shows that the physicians composing the faculties of the two schools recognised the needs of modern medicine and were willing to make sacrifices in the interest of higher medical education.

THE New York Post-Graduate Hospital was endangered by fire on Friday, October 11, 1907, which caught in the storeroom, located in the basement of the great building. It arose from the defective insulation of electric wires. The patients were brought down from the upper floors, although the members of the house staff had put out the fire with hand extinguishers before the firemen reached the scene.

THE Governors of the New York Skin and Cancer Hospital announce that Dr. L. Duncan Bulkley will give a ninth series of Clinical Lectures on Diseases of the Skin, in the out-patient hall of the hospital on Wednesday afternoons from November 6 to December 18, 1907, at 4.15 o'clock. The course will be free to the medical profession.

